# Detecting Hepatitis B Surface Antigen Mutants

**DOI:** 10.3201/eid1202.050038

**Published:** 2006-02

**Authors:** Paul F. Coleman

**Affiliations:** * Abbott Laboratories, Abbott Park, Illinois, USA

**Keywords:** Hepatitis B virus, HBsAg, mutations, immunoassay, perspective

## Abstract

Hepatitis B viral mutants can emerge in patients as a result of selection pressure from either immune response or treatment options. Mutations that occur within the immunodominant epitopes of hepatitis B surface antigen (HBsAg) allow mutant virus to propagate in the presence of a neutralizing immune response, while wild-type virus is reduced to undetectable levels. HBsAg mutants present as false-negative results in some immunoassays. An understanding of immunoassay reactivity with HBsAg mutants is key to establishing an appropriate testing algorithm for hepatitis B virus detection programs.

## Abstract 2

The emergence of HBsAg mutants presents a challenge to HBV screening programs.

Over the past decade, the importance of hepatitis B virus (HBV) mutants has made a transition from an academic phenomenon of unknown prevalence to a factor for consideration during disease diagnosis. HBV infection has a major effect on world health care: more than one third of the world's population has been infected at some point; ≈350 million people are currently infected (*1*). This immense worldwide reservoir of infection serves as the basis for the generation of HBV mutants because of the unique molecular biology of this virus. Since the late l980s, we have seen the emergence of mutants across the entire HBV genome as the virus responds to selective pressures, such as vaccination and antiviral therapy. Viral adaptation through mutation will continue as new treatment options are employed and current treatment options are expanded into areas of endemic infection. HBV mutant surveillance and understanding of HBV mutant impact on disease diagnosis will pose a challenge to global health care for the foreseeable future. Thus, diagnosticians and the healthcare industry need to increase their awareness of HBV mutants and how these mutants may alter current diagnostic and treatment algorithms. This article addresses recent information concerning the emergence of hepatitis B surface antigen (HBsAg) mutants, their impact on viral antigen presentation, latest prevalence data, and discussion of the issues associated with detection of mutants in healthcare settings.

## Mechanism of HBV Mutant Generation

HBV belongs to the genus *Orthohepadnavirus*, family *Hepadnaviridae*. This virus has a small circular DNA genome, ≈3.2 kb in length, that contains 4 genes with partially overlapping open reading frames (ORFs). These ORFs encode the polymerase protein (Pol gene); core antigen and e antigen (C gene); large, medium, and small surface-antigen proteins (S gene); and the X protein (X gene). From a relatively small genome, these overlapping ORFs generate 7 proteins. While this gene overlap may constrain some viral variability, mutant or variant forms have been identified for all 4 genes (*2*). HBV analysis has transitioned from the serologic subtype classification of the early 1970s to the more precise genotype genetic classification. HBV has been classified into 8 genotypes (A–H) on the basis of intergenotypic difference of >8% in the entire nucleotide sequence (*3*). HBV genotypes demonstrate geographic diversity. However, distinct genotypes have evolved in more remote areas, as evidenced by genotype E, localized in Madagascar, and genotype F, localized in South America. This diversity of the HBV genome is generated by the same mechanism that drives the emergence of mutants, replication.

The replication of HBV DNA proceeds through a RNA reverse transcriptase intermediary step. HBV variants are generated during this process. Since the reverse transcriptase activity of the HBV polymerase protein lacks a proofreading function, random mis-incorporation of bases into the replicating DNA strand occurs. This mismatch leads to the generation of multiple variant transcripts from a single template and the formation of a quasispecies pool (*4*). This quasispecies pool provides the source material for the emergence of a mutant when selection pressure is applied (*5*). A mutation selected in 1 gene can potentially lead to an amino acid change in the overlapping reading frame. Replication of the hepatitis B virion is, therefore, the sole requirement for generating these nucleotide mismatch sequences. The number of viral particles generated in some infected persons can be as high as 10^11^ viral particles per day. Because of the polymerase reverse transcription error rate (1 error per 10^7^ bases), in active infection, 10^7^ base-pairing errors can be generated per day over the 3,200-bp genome (*6*). While most of these new sequences are nonviable or fail to effectively compete with wild-type virus, they provide a starting point for the emergence of mutants when selection pressure is applied. HBV mutants can be expected to emerge in any geographic area where populations of infected persons are exposed to a selective pressure.

New treatment regimens developed over the past 2 decades have successfully reduced overall HBV infection rates, but they have also exerted powerful selection pressures for the emergence of HBV mutants. Treatments that have selected for mutants include immunotherapy (vaccination, administration of HBV immune globulin) and nucleoside analogs (e.g., lamivudine, lobucavir, famciclovir, adefovir) to inhibit polymerase activity. These treatment options can suppress wild-type HBV to undetectable levels, allowing a mutant HBV strain to emerge as the predominant form. Emergence of a mutant species can be monitored by using such techniques as real-time polymerase chain reaction (PCR) assays, restriction length polymorphism assays, quantitative fragment analysis, and primer extension assays. These powerful techniques can detect trace mutant sequences in clinical samples with a preponderance of wild-type virus, while conventional DNA sequencing cannot (*7*). Mixed infection samples (i.e., low-level HBV chronic infections) that contain a preponderance of wild-type HBsAg present a challenge to immunoassay sensitivity, not epitope recognition. We only address the detection of HBsAg mutants in clinical samples that appear to be homogenous and therefore specifically challenge immunoassay epitope recognition. Replication-defective mutants, intracellular accumulation of normally secreted antigens, and tissue localization can also affect mutant detection in clinical samples.

## Surface Antigen Structure

The translational products of the surface antigen gene consist of 3 proteins that have different initiation sites with the same termination site. The most important of these proteins, from a diagnostic standpoint, is the small HBsAg (sHBsAg) protein, which is composed of 226 amino acids (aa). sHBsAg is the major structural protein of the hepatitis B viral envelope. Most HBsAg in the plasma of HBV-infected persons consists of 22-nm spherical particles composed of ≈100 HBsAg monomers each (*8*). Initial studies noted that HBsAg has a complex structure with discontinuous epitopes. The possibility of multiple antigenic conformations or intermolecular epitopes cannot be ruled out when considering surface antigen structure. This antigenic complexity has impeded elucidation of HBsAg structure.

The HBsAg amino acid sequence contains a highly conformational, hydrophilic domain from positions 100 to 160 referred to as the "a" determinant. The "a" determinant represents the immunodominant region of HBsAg. The reagents used in many HBsAg diagnostic assays are directed against epitopes in the "a" determinant. The "a" determinant conformational epitopes are stabilized by a backbone of conserved disulfide-bonded cysteine residues. Alteration of residues in the "a" determinant can result in reduced antigenicity and reduced levels of protein expression (*9*). Using a combination of conformational peptides (*10*) and phage display experiments (*11*), we constructed a working model of the "a" determinant (Figure). The key features of this model include a large laminar loop stabilized by bonding between cysteine residues 108–138 with a fingerlike projection stabilized by disulfide-bonded 121–124 cysteine residues. While other cysteine residues affect antigenicity when mutated, a double mutation of these 121–124 cysteine residues has physical properties similar to those of wild-type virus (*12*). These data indicate that the fingerlike projection at aa 121–124 forms an epitope that is relatively isolated from other substitutions in the "a" determinant. The model also includes a second loop, which projects from the viral membrane and is stabilized by bonding between cysteine pairs 136–149 and 139–147. The human immune response to HBsAg is primarily directed against disulfide-bonded conformational epitopes of the "a" determinant and can be classified into a limited number of epitopes (*13**–**15*). Alteration of these conformational epitopes not only can result in failure to neutralize viral infection but also can affect diagnostic assay detection, depending on the epitopes recognized by the assay reagent configuration.

**Figure:**
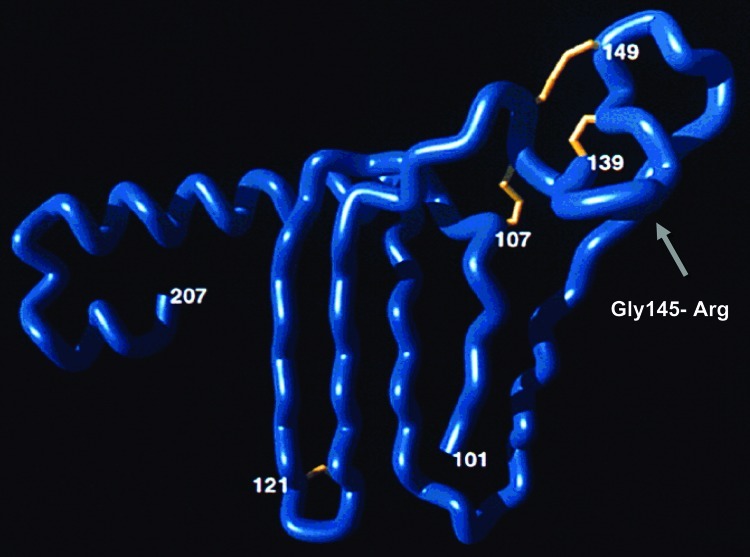
Gly/Arg 145 mutant in the projecting amino acid 139–147 antigenic loop of the "a" determinant. This mutant produces false-negative results in some commercial assays. Image courtesy of Y.C. Chen et al. (*11*).

## Surface Antigen Mutants

The initial description of an HBsAg mutant was made in the breakthrough infection of a child born to a HBV-positive mother (*16*). The virus was vertically transmitted despite the child's being vaccinated and passively immunized against HBV. The breakthrough viral strain was DNA sequenced and shown to contain a substitution mutation of glycine to arginine at HBsAg aa position 145 (Gly/Arg 145) (*17*). The child subsequently remained both DNA- and HBsAg-positive for this Gly/Arg 145 mutant for >12 years, despite having protective antibody to surface antigen (anti-HBs) titer against the wild-type virus. The Gly/Arg 145 substitution alters the projecting loop (aa 139–147) of the "a" determinant such that neutralizing antibody induced by vaccination no longer recognizes the mutated epitope, hence the term vaccine-escape mutant. Wild-type HBsAg is reduced to undetectable levels in these patient samples. For the vaccine-escape mutant to emerge, the patient's anti-HBs response must be localized to the aa 139–147 region; the Gly/Arg 145 substitution thus confers a selective advantage in viral replication, and the mutant becomes the dominant form of the virus (*18*). The replication of Gly/Arg 145 mutants has been investigated with chimpanzee infection models. In the first study, a wild-type HBV infection developed in chimpanzees inoculated with a human sample of Gly/Arg 145 HBV; only samples diluted >10^–6^ established mutant infection (*19*). Since the pol gene ORF partially overlaps the S gene, the Gly/Arg 145 mutation in the S gene sequence corresponds to a Trp/Gln 153 mutation in the pol gene sequence, which results in the expression of an altered polymerase gene product. This altered polymerase is replication competent but has reduced replication efficiency (*6*). When anti-HBs selection pressure is removed, wild-type HBV returns as the predominant infectious form because of the impeded replication of the Gly/Arg 145 mutant. These facts may explain why transmission studies have failed to show mutant transmission to vaccinated animals (*20*). If the recipient animal had an anti-HBs response directed against an epitope outside the aa 139–147 region, the mutant inoculum would be neutralized by anti-HBs binding to epitopes unaffected by the Gly/Arg 145 escape mutation. In this case, no HBV infection would be established. Since the emergence of the Gly/Arg 145 mutant is constrained by requiring the host antibody response to be directed solely against the aa 139–147 region, whether the Gly/Arg 145 mutant will become the predominant infectious form of HBV in the future, as some models have predicted (*21*), is questionable.

The Gly/Arg 145 substitution remains by far the predominant HBsAg mutant described in the literature (*22*). However, a wide range of mutants have been described in the past 10 years, including many amino acid substitution mutants across the "a" determinant (*23*), amino acid insertions into the "a" determinant (*24**,**25*), and deletion mutants (*7**,**26*). Some of these substitution mutants appear to be of academic interest as they occur at very low levels in long-term HBV carriers and have only been identified by highly directed DNA amplification techniques that used primers specific for mutant sequence detection. The conditions for performing highly amplified PCRs must include controls to ensure that any sequence changes found are not an artifact of PCR fidelity itself (*27*). Some HBV isolates found in screening studies may be infrequently occurring natural variants (*28*). Given the diversity of HBV genotypes, the categorization of a novel HBsAg amino acid change as a mutant should hinge on a tangible alteration in viral function, such as antigenicity, infectivity, replication, and morphology, which is attributable to the specific change. One method for establishing a mutant is to introduce the suspected amino acid change into a wild-type backbone sequence and demonstrate altered function.

Important to the healthcare management of HBV infection is detection of HBsAg mutants by diagnostic assays. HBsAg is a sentinel marker in blood bank donor screening to prevent transmission of HBV infection in patients receiving transfusions. A diagnostic assay used for HBV screening may show false-negative results if the assay configuration cannot detect mutants in the "a" determinant. Initial reactivity data on 9 HBsAg assay configurations determined for 28 defined and quantitated HBsAg recombinant mutant antigens (*29*) have been confirmed by several groups. In these studies, recombinant HBsAg antigens containing a single amino acid substitution in an otherwise wild-type sequence were tested for immunoassay reactivity. Since the level of protein expression varies greatly for each recombinant HBsAg mutation, diluting each recombinant mutant protein to a known concentration before immunoassay testing was important. By setting the concentration of each recombinant mutant sample well above the antigen endpoint detection of the assays tested, the possibility of false-negative results caused by assay sensitivity was eliminated. Therefore, false-negative results were due to failure to detect the mutated epitopes of the recombinant antigen. Recombinant HBsAg that represents common mutants found in neonatal breakthrough infections was tested with different immunoassay formats (Table 1). Substitution mutants in the projecting loop of the aa 139–147 region were not detected by some commercial assays. Later generation HBsAg assays have enhanced reagent configurations that allow them to detect not only the common HBsAg mutants but also the rare mutations that occur in the aa 121–124 region such as the Arg + Ala 123 insertion mutant (*29*). This mutant produces a 228-aa surface antigen (instead of the wild-type 226 aa antigen) with gross alteration of "a" determinant epitopes. This mutant is one of the most challenging to detect by an immunoassay format. In addition to the recombinant antigens, 3 corresponding patient samples containing the native HBsAg mutants were also available for testing. The data indicated that the immunoreactivity of both the recombinant antigen and the original patient sample were the same. No wild-type antigen was detectable in the original patient samples. Not quantitating recombinant HBsAg mutant antigens before immunoassay evaluation can account for some conflicting immunoassay detection results published in subsequent studies (*30*). Moerman et al. (*31*) have recently published an expanded selection of immunoassays and their detection of the more common HBsAg mutants (Table 2). Four commercially available assays were tested with both recombinant antigens containing defined mutations within the "a" determinant (samples 1–10) and with actual serum samples containing HBsAg mutants (samples 11–14). Several assays detected all of the mutant panel members, while others failed to detect >1 panel member. The detection of recombinant antigens paralleled the detection of patient serum samples. Furthermore, only mutant HBsAg appears in the false-negative clinical samples, as wild-type antigen would have been detected by the corresponding assays if present at sufficient levels. Other investigators have also confirmed the findings that some immunoassays are susceptible to the common "a" determinant mutants and produce false-negative results (*32*).

**Table 1:** Detection of most common hepatitis B surface antigen (HBsAg) mutants by 9 commercial assays (*29*)*†

Assay configuration		Ausria poly/poly	Auszyme mono/mono	IMx HBsAg mono/poly	AxSYM HBsAg mono/poly	PRISM HBsAg mono/poly	ARCHITECT HBsAg mono/poly	Commercial assay A mono/mono	Commercial assay B mono/mono	Commercial assay C poly/mono
HBsAg mutants										
	Wildtype	+ +	+ +	+ +	+ +	+ +	+ +	+ +	+ +	+ +
	Thr126- Ser	+ +	+	+	+ +	+ +	+ +	+	+ +	+ +
	Gln129- His	+ +	+	+	+ +	+ +	+ +	+	+ +	+ +
	Met133- Leu	+ +	+ +	+ +	+ +	+ +	+ +	+ +	+ +	+ +
	Asp144- Ala	+ +	+ +	+ +	+ +	+ +	+ +	–	+ +	+ +
	Gly145- Arg	+ +	+ +	+ +	+ +	+ +	+ +	–	–	–
	Thr126- Ser + Gly145- Arg	+ +	+ +	+	+ +	+ +	+ +	–	–	–
	Pro142- Leu + Gly145- Arg	+ +	+ +	+ +	+ +	+ +	+ +	–	–	–
	Pro142- Ser + Gly145- Arg	+ +	+ +	+ +	+ +	+ +	+ +	–	–	–
	Asp144- Ala + Gly145- Arg	+ +	+ +	+ +	+ +	+ +	+	–	–	–

*All positive samples confirmed in their respective assays. †+ +, equivalent detection to wild-type antigen; +, detection less than wild-type antigen; –, not detected.

**Table 2:** Detection of hepatitis B surface antigen (HBsAg) mutants by 4 commercial assays (*30*)

Capture/detection		Abbott AxSYM HBsAg mono/poly	Bayer Centaur HBsAg mono/mono	Ortho Vitros ECi HBsAg mono/mono	Roche Elecsys HBsAg mono/mono
Recombinant samples		(s/co)	(s/co)	(s/co)	(s/co)
	Wild-type	5.45	15.17	15.95	9.77
	Thr126- Ser	4.74	20.17	12.50	8.64
	Gln129- His	4.48	20.12	13.85	8.61
	Met133- Leu	4.84	12.72	12.15	8.58
	Asp144- Ala	3.65	6.47	0.12	6.55
	Gly145- Arg	3.85	<0.10	0.06	0.56
	Thr126- Ser + Gly145- Arg	3.36	<0.10	0.05	0.51
	Pro142- Leu + Gly145- Arg	3.77	<0.10	0.05	0.55
	Pro142- Ser + Gly145- Arg	4.08	<0.10	0.06	0.54
	Asp144- Ala + Gly145- Arg	3.62	<0.10	0.06	0.52
Clinical samples					
	Gly145 - Arg	5.85	<0.10	0.40	0.70
	Pro120-Gln/Thr-131Lys/Gly145-Arg	2.48	<0.10	0.14	0.62
	Thr118-Val/Met133-Ile/Phe134-Asn/Pro142-Ser/Thr143-Leu/Gly145-Arg	17.60	<0.10	0.11	0.67
	Thr115-Asn/Pro120-Leu/Met133-Ile/Phe134-His/Asp144-Val/Ser154-Pro	2.73	<0.10	0.10	0.58

Case reports of false-negative diagnostic results due to HBsAg mutants have been described in blood bank (*33*) and hospital settings (*34*). The blood bank sample is of special importance since this patient sample (containing a Thr/Leu 143 mutant) was reported as HBsAg positive by 1 screening immunoassay, while a second screening immunoassay reported the same sample as false-negative. The Thr/Leu 143 mutant may be more prevalent than originally thought, as another occurrence has been recently reported in Europe (*35*). Screening efforts should be undertaken to establish the prevalence of this apparently emerging mutant and to establish its mechanism of selection.

In most cases, investigators reporting false-negative results due to HBsAg mutants recommend that laboratory users of HBsAg assays be aware of a given assay's ability to detect mutants. An expert advisory meeting has recently issued a consensus report on emerging HBsAg mutants (*36*). The meeting participants concluded that the prevalence of HBsAg mutants is probably higher than previously believed. The participants called for enhanced surveillance efforts and data collection for mutants and recommended using assays that detect the most frequently observed mutants at aa positions 139–145. In addition, users should develop an appropriate testing and confirmatory algorithm to ensure mutant detection. The prevalence of HBsAg mutants can be established in laboratories that perform sequential testing of a sample using 2 assays, each with differing susceptibility to mutant false-negative results. Discordant positive samples would be PCR amplified and sequenced to determine if a mutant sequence is present. In a study in Singapore, the Gly/Arg 145 mutation was present alone or in combination with other mutations in 70% of the isolated HBsAg mutants from neonatal breakthrough infections, for an overall mutant prevalence of 4.6% in this population (*37*). A screening program for school-age children in Taiwan found 27/3,849 patient samples with "a" determinant mutants for a prevalence of 0.7% (*38*). In India, testing of an HBV chronic carrier's household contacts found what might be the first documented case of Gly/Arg 145 horizontal transmission (*39*). Therefore, the Gly/Arg 145 mutant occurs at a significant rate in some populations and appears to be horizontally transmissible, which suggests that HBV surveillance programs should use diagnostic methods capable of detecting this mutant.

In contrast, substitutions at positions outside of the "a" determinant appear to be readily detected by current commercially available HBsAg immunoassays. For example, mutations near the carboxy terminus of the small HBsAg protein occur when polymerase mutations are selected for in the YMDD reverse transcriptase domain (again well outside the "a" determinant). Of greater interest are the secondary compensatory changes emerging in polymerase mutants (*6*). These "polymerase stabilizing" mutations are expressed in HBsAg close to or in the "a" determinant and reduce HBsAg immunoreactivity (*40*). The risk of a "stabilized" polymerase mutant with altered HBsAg epitopes (presumably from a patient on long-term nucleoside analog treatment) being transmitted to a compatible recipient is a key issue for diagnosticians to monitor in the future. These mutants would potentially produce false-negative test results in susceptible HBsAg immunoassays and yet have the capacity to replicate in a manner similar to that of wild-type virus. Reporting mutant occurrence at the national level by using data-tracking to monitor regional exposure would mitigate such a risk.

These studies of recombinant surface antigen mutants underscore the usefulness of mapping the epitope susceptibility of various commercially available HBsAg assays. While testing of mutant panels is voluntary in some countries, certain regulatory agencies are becoming increasingly aware of HBsAg mutants. In the United States, manufacturers of new HBsAg assays must address mutant detection in their package inserts. With a firm understanding of immunoassay mutant detection, the diagnostician can select the appropriate HBsAg screening algorithm to minimize the impact of mutants in sentinel screening programs.
